# Body Composition and Cardiovascular Risk: A Study of Polish Military Flying Personnel

**DOI:** 10.3390/metabo13101102

**Published:** 2023-10-21

**Authors:** Agata Gaździńska, Stefan Gaździński, Paweł Jagielski, Paweł Kler

**Affiliations:** 1 Laboratory of Dietetics and Obesity Treatment, Department of Psychophysiological Measurements and Human Factor Research, Military Institute of Aviation Medicine, Krasińskiego 54/56, 01-755 Warsaw, Poland; 2 Department of Neuroimaging, Military Institute of Aviation Medicine, Krasińskiego 54/56, 01-755 Warsaw, Poland; sgazdzin@wiml.waw.pl; 3 Department of Nutrition and Drug Research, Faculty of Health Science, Jagiellonian University, Medical College, Skawińska 8, 31-066 Cracow, Poland; paweljan.jagielski@uj.edu.pl; 4 Department of Security, Logistics and Management, Institute of Logistics, Jarosław Dąbrowski Military University of Technology, Gen. Sylwestra Kaliskiego 2B, 00-908 Warsaw, Poland; pawel.kler@wat.edu.pl

**Keywords:** obesity, cardiovascular disease, soldiers, military flying personnel, Polish Army, national security

## Abstract

(1) Background: Military personnel worldwide exhibit high rates of obesity. Obesity, and especially visceral obesity, contribute to various health issues, including type 2 diabetes and cardiovascular diseases (CVD). While BMI is commonly used to diagnose obesity, it has limitations and does not consider factors like fat distribution or muscle mass. This study aims to assess the relationship between BMI, percent body fat, waist circumference (WC), waist-to-height ratio (WHtR), and cardiovascular risk factors in Polish military flying personnel. Methods: This study involved 200 men from the Polish Air Force aged 38.8 ± 8.5 years. Anthropometric tests, body composition tests, and tests of biochemical markers of CVD were conducted. (2) Results: The prevalence of overweight and obesity varied based on the evaluation criterion; they were present in 63.5% of soldiers by BMI and in 52.5% by percent body fat; abdominal obesity was present in almost half (47%) of the surveyed soldiers according to WC and in 62.5% according to WHtR. All markers of obesity correlated positively with various biochemical markers of CVD, and 8.5% of subjects met the criteria for metabolic syndrome. (3) Conclusions: The prevalence of obesity in Polish military flying personnel, regardless of the evaluation criterion, is associated with significant metabolic complications in the form of lipid disorders and insulin resistance.

## 1. Introduction

Obesity is recognized by the World Health Organization (WHO) as an epidemic of the 21st century. It is considered a major public health problem and the fifth most important cause of death worldwide [1]. Studies have shown an alarmingly high prevalence of excessive body weight among soldiers all over the world. Soldiers fulfilling the criteria of obesity constitute 8% in the US Army [2], 10% in the French Army [3], 12% in the British Army [4], 13% in the Iranian Army (13%) [5], and from 3% among cadets [6] to 17% among soldiers in the Polish Army [6,7,8]. An even higher percentage of soldiers with excessive body weight was found in the Saudi Arabian Army, where obesity was diagnosed in 44% of soldiers [9]. Excessive weight in soldiers can lead to the development of many dangerous diseases that can affect national security. In our earlier manuscript, we demonstrated that among Polish soldiers, the main risk factors for obesity were age over 40 years, too little sleep (less than 6 h), reaching for food in stressful situations, having an obese mother, not exercising or exercising at most two days a week, and spending two or more hours a day in front of the television [7].

Obesity, especially visceral obesity, contributes to the development of type 2 diabetes, insulin resistance, cancer, osteoarthritis, sleep apnea, and cardiovascular disease (CVD), such as hypertension, coronary heart disease, heart failure, supraventricular ventricular arrhythmias, and dyslipidemia [10,11]. It also increases the risk of sudden cardiac death [12,13,14].

Adipokine interactions, insulin resistance (IR), chronic inflammation, and increased blood clotting are known to contribute to the development of cardiovascular disease in obese individuals [15,16]. IR can promote atherogenesis and CVD via multiple mechanisms, including changes in classic CVD risk factors and downregulation of the insulin signaling pathways in different target tissues [17]. It is widely accepted that professional soldiers should not have CVD. However, even with careful selection for physical fitness and overall health, they are not free from CVD risk factors [18,19]. Metabolic effects of visceral fat and adipose tissue located adjacent to the heart and blood vessels also play an important role [20,21]. Additionally, the current literature highlights the positive effects of ghrelin on the cardiovascular system, as it improves endothelial function by increasing the bioavailability of nitric oxide [22].

In diagnosing obesity, the most commonly used marker is the body mass index (BMI), which is calculated by dividing body weight (kg) by height squared (m^2^), according to WHO criteria [23] (accessed on 20 March 2023). Overweight is diagnosed when the BMI is between 25 and 29.9 kg/m^2^ (the so-called pre-obesity state), and obesity is diagnosed with a BMI ≥ 30 kg/m^2^. While this index is widely used around the world because of its simplicity of use, it has many limitations. The interpretation of BMI values in adults does not depend on age or gender, and it does not reflect the content or distribution of body fat (subcutaneous, visceral), the ratio of body fat to muscle tissue, or hydration status. It is well known that the effect of increased BMI may be related to expanded muscle mass (so-called muscular overweight), which is relatively common among soldiers [24,25,26]. Taken together, the determination of BMI alone is insufficient to estimate the impact of obesity on health. The other measures used to determine obesity are waist circumference (WC) and waist–hip ratio (WHR); they are used to discriminate visceral adiposity from simple obesity [27]. Nonetheless, WC does not account for differences in height, and it could thus lead to overestimation or underestimation of risk for tall and short individuals, respectively. Moreover, the WHR might be inaccurate in persons who have lost weight [28].

The waist-to-height ratio (WHtR) is an alternative measurement of visceral fat. A systematic review published in 2010 concluded that WHtR may be advantageous because it avoids the need for age-, sex-, and ethnicity-specific values [28]. Higher WHtR values ≥ 0.5 indicate a higher risk of obesity-related diseases (mainly related to abdominal obesity) [29]. WHtR, like waist circumference, correlates strongly with abdominal fat content, as measured by imaging methods [30]. It has also been shown that there is not so much an influence but a correlation of this index with the risk of other diseases and risks, such as myocardial infarction, stroke, and mortality from ischemic heart disease. Values ≥ 0.5 indicate an increased risk of cardiovascular disease and diabetes [27,31]. In longitudinal studies, this indicator better illustrates the risk of morbidity and mortality than BMI [32].

Hence, the aim of the presented study was to evaluate the relationship between BMI, body fat content, waist circumference, and waist-to-height ratio and parameters of carbohydrate, lipid, and hormone metabolism, as well as insulin resistance, which are cardiovascular risk factors in Polish military flying personnel. Additionally, we evaluated to what degree the method used to determine overweight and obesity affects the values of the cardiovascular risk factors.

## 2. Materials and Methods

### 2.1. Participants

This study included 200 men (mean age 37.8 ± 8.5 years) who are members of the active military flying personnel of the Polish Air Force who took part in the National Health Programme 2016–2020 Project. They were individuals who consecutively reported for mandatory annual anthropometric examinations to the Laboratory of Dietetics and Obesity Treatment as part of routine adjudicatory and medical examinations at the Military Institute of Aviation Medicine in Warsaw, Poland. They expressed a desire to participate in additional examinations carried out as part of the National Health Program 2016–2020. All of them had current fitness to fly certificates given by the Aeromedical Board (i.e., they were healthy). All procedures were conducted in September–November 2018. All procedures were approved by the Institutional Review Board of the Military Institute of Aviation Medicine, Warsaw, Poland (decision No. 01/2018 of 9 March 2018), and this study was performed in accordance with the ethical standards as laid down in the 1964 Declaration of Helsinki and its later amendments or comparable ethical standards. All participants signed informed consent forms.

### 2.2. Assessment of Nutritional Status

The degree of overweight and obesity was assessed according to the body mass index (BMI) according to the World Health Organization (WHO) criteria [23] (accessed on 20 March 2023). Body mass, percent body fat content (PBF) (defined as the percent of contribution of fat mass to the mass of the body), fat mass, muscle mass, and fat-free mass were measured using the bioelectrical impedance method with the Inbody 370 analyzer (InBody, Tokyo, Japan). 

According to a publication by Gallagher et al. [33], 8–20% body fat content in men was considered the normal and optimal range. Underweight was diagnosed at a body fat content of less than 8%, and overweight was diagnosed at 21–25% body fat content. The body fat content characteristic of obesity was diagnosed at values above 25%. Waist circumference (WC) was measured with a non-stretchable standard tape over the unclothed abdomen at the smallest diameter between the costal margin and the iliac crest, according to International Diabetes Federation guidelines; abdominal obesity was diagnosed in subjects when the waist circumference (WC) was ≥94 cm [34]. However, in the 2001 National Cholesterol Education Program Adult Treatment Panel III (NCEP ATP III) guidelines, abdominal obesity was defined as a WC ≥102 cm in males [35]. It should be noted that in many countries there are three stages of abdominal obesity: 94–102 cm and >102cm for abdominal obesity [36]. 

The waist-to-height ratio (WHtR) was also calculated. WHtR is defined as waist circumference divided by height; body height was measured with a Harpenden Anthropometer (Holtain Ltd., Crosswell, Crymych, UK) to the nearest 1 mm in a standing, upright position without shoes. Physical measurements, including weight, standing height, and the circumferences of the waist and hips, were carried out according to standard protocol by trained assistants. Smoking habits were determined by self-questionnaires.

### 2.3. Laboratory Tests

All examinations were performed on soldiers after an overnight fast. Blood samples were taken in the morning around 7 o’clock. Concentrations of the following biochemical markers were determined using standard methods:

- Total cholesterol (TC) (enzymatic-colorimetric method using a Cobas Integra 400 plus analyzer (Roche Diagnostics, Warsaw, Poland). Values < 190 mg/dl were taken as the norm.
- Triglycerides (TG) (enzyme-colorimetric method using a Cobas Integra 400 plus analyzer). Values of ≤150 mg/dl were taken as the norm.
- Low-density lipoproteins (LDL-C) (parameter calculated according to the Friedewald formula). Values < 115 mg/dl were taken as the norm.
- High-density lipoproteins (HDL-C) (enzyme-colorimetric method using a Cobas Integra 400 plus analyzer).
- Glucose (enzymatic method with hexokinase using a Cobas Integra 400 plus analyzer). Values < 99 mg/dl were taken as the norm.
- Insulin (ECLIA electrochemiluminescence method using a Cobas e 411 analyzer). Values of 2.6–24.9 µIU/mL were taken as the norm.
- Ghrelin (ELISA method using an ETI-Max 3000 analyzer; DiaSorin S.p.A., Saluggia, Italia).

Insulin resistance was diagnosed based on the insulin resistance index Homa-IR (Homeostatic Model Assessment) using the following formula [37]:

HOMA = [fasting insulinemia (mU/mL) × fasting blood glucose (mmol/L)]/22.5, using ≥ 2.5 as cutoff values

Blood pressure was checked by an internal medicine physician. All of the studied soldiers had blood pressures within the normal range. Nevertheless, it should be noted that we cannot exclude cases of treating hypertension outside the military health service and not informing the air medical board of this fact for fear of not receiving a positive opinion from the medical examiner. 

Based on the results of these tests, the percentage of soldiers meeting the criteria for metabolic syndrome (waist circumference ≥ 102 cm or BMI ≥ 30 kg/m^2^ and, at the same time, fasting glucose ≥ 100 mg/dl and non-HDL cholesterol ≥ 130 mg/dl, where non-HDL cholesterol = total cholesterol − HDL cholesterol) [38] was calculated.

The results of the above tests were compared according to the eligibility criteria for overweight and obesity, both by (1) BMI, (2) body fat percentage, and (3) waist circumference. In addition, the results of laboratory tests were compared with reference ranges and presented as the percentage of subjects with out-of-normal parameters, according to BMI and percent body fat. The latter is due to the fact that the effect of increased BMI may be related to expanded muscle mass (so-called muscular overweight), which is relatively common among soldiers [24,25,26].

### 2.4. Statistical Analysis

The results for quantitative variables were presented in the form of descriptive statistics (abundance, mean, standard deviation, and median) and, for qualitative variables, in the form of frequencies and graphs. For comparative analyses between groups (nutritional status by BMI, body fat, and waist circumference), chi^2^ and Kruskal–Wallis tests were used. If the Kruskal–Wallis test confirmed differences between groups, multiple comparisons of mean ranks were then used for all groups. Relationships between variables were checked using the Spearman correlation. A statistical significance value of *p* < 0.05 was assumed, and the normality of distributions was checked using the Shapiro–Wilk test. All analyses were performed using STATISTICA version 13.0 PL software.

## 3. Results

The main demographic variables are presented in Table 1. Among the subjects, 10.5% declared that they smoked tobacco. The criteria for metabolic syndrome in the study group were met by 17 soldiers (8.5%).

**Figure 1:**
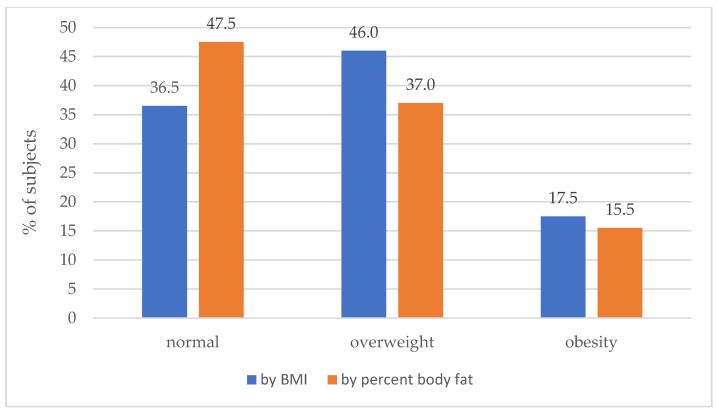
Proportions of normal-weight, overweight, and obese individuals according to the assessment method (here, BMI vs. percent body fat) in surveyed military flying personnel. Please note lower rates of overweight and obesity according to percent body fat compared to BMI, likely due to larger muscle mass in soldiers than in the general population (so-called muscular overweight).

As expected, the prevalence of overweight and obesity in the surveyed soldiers differed according to the evaluation criterion (see Figure 1). As an illustration, the number of soldiers at normal weight was underestimated by about 30%; it is the relative difference in prevalence of normal weight, when using BMI (36.5%) and percent body fat content (47.5%), scaled to the prevalence of normal weight for BMI (36.5%), in percent.

**Figure 2:**
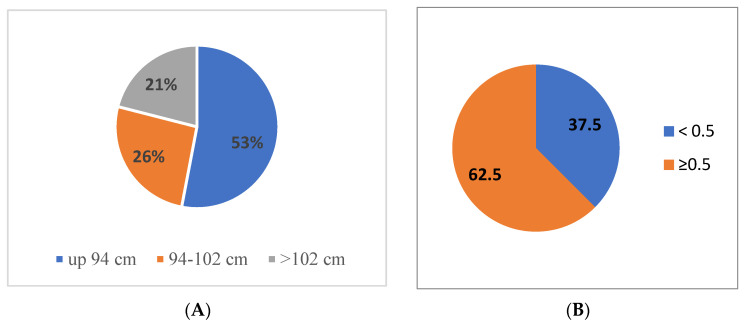
Nutritional status categories according to waist circumference (**A**) and waist-to-height ratio (WHtR) (**B**) among the surveyed military flying personnel.

Abdominal obesity, on the other hand, was present in almost half (47%) of the surveyed soldiers according to WC, and in 62.5% according to WHtR (Figure 2). However, it should be noted that the personnel with diagnosed obesity according to BMI were significantly older (*p* < 0.001) compared to overweight and normal weight subjects. This relationship was not observed between groups defined according to percent body fat (*p* > 0.05) (Table 2).

Soldiers with diagnosed obesity according to BMI had significantly higher scores on all body composition and anthropometric parameters (Table 1) than those with normal BMI values. The mean body fat content in obese subjects was 29% vs. 17% in normal-weight subjects (*p* < 0.001). In the obese soldiers, compared to the remaining soldiers, both average waist circumference (107 cm vs. 84 cm) (*p* < 0.001) and the WHtR were higher (0.47 vs. 0.60, *p* < 0.001).

The comparative analysis of total body water content, lean body mass, and skeletal muscle mass in the groups of soldiers divided according to body fat content did not show any significant differences (*p* > 0.05; see Table 2). However, the other parameters studied were significantly higher in soldiers with body fat content indicating obesity compared to those with normal body fat content (*p* < 0.001). 

The results of laboratory blood tests of military flying personnel divided into groups according to BMI, body fat content, and waist circumference are shown in Table 3, Table 4, and Table 5, respectively. High BMI and body fatness translated directly into laboratory test results. Individuals diagnosed with obesity according to BMI were characterized by significantly higher blood concentrations of such biochemical parameters as TC, TG, LDL-C and HDL-C, insulin, and HOMA-IR index compared to those with normal BMI. Similar observations were noted when analyzing the blood results of soldiers divided into groups based on percent body fat (Table 4), except for LDL-C, where no significant differences were noted (*p* > 0.05). 

The statistical analysis showed no significant differences between the ghrelin and blood glucose concentrations of the soldiers studied in groups divided by BMI and body fat content (Table 3 and Table 4). Significant differences in blood glucose concentrations were noted only between groups divided according to waist circumference (Table 5). Those with a circumference of more than 94 cm had significantly higher concentrations of this parameter than the remaining participants (*p* < 0.001).

BMI, percent total body fat, waist circumference, and WHtR of the soldiers studied were positively correlated with each other, as well as with the concentrations of glucose, TC, TG, LDL-C, insulin, and HOMA-IR index, but they were negatively correlated with blood HDL-C concentration (*p* < 0.05). Nutritional status was not related to ghrelin concentrations in the blood of the subjects (Table 6).

**Figure 3:**
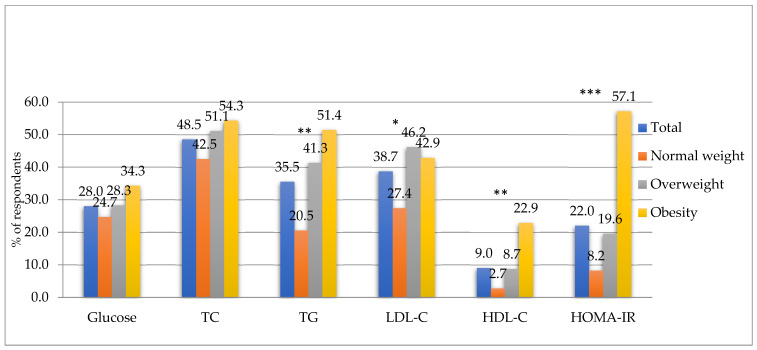
Percentage of subjects with out-of-normal blood parameters according to BMI. TC—total cholesterol, TG—triglyceride, LDL-C—low-density lipoprotein cholesterol, HDL-C—high-density lipoprotein cholesterol. *** *p* < 0.001, ** *p* < 0.01, * *p* < 0.05, *p*—chi-square test statistical significance value.

**Figure 4:**
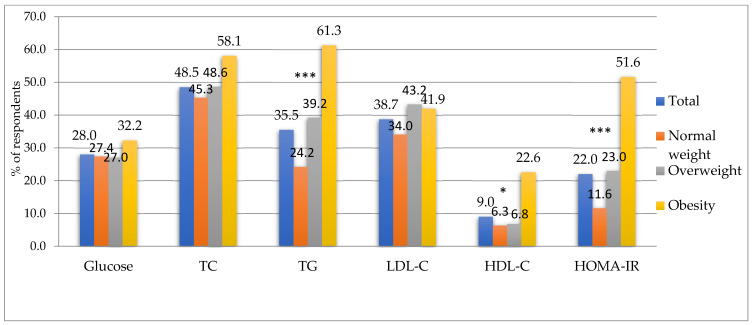
Percentage of subjects with out-of-normal blood parameters according to body fat content. TC—Total cholesterol, TG—triglyceride, LDL-C—low-density lipoprotein cholesterol, HDL-C—high-density lipoprotein cholesterol, *** *p* < 0.001, * *p* < 0.05, *p*—chi-square test statistical significance value.

The percentage of surveyed soldiers with parameters outside the normal range according to BMI is shown in Figure 3, while the results according to percentage body fat content are shown in Figure 4, respectively. It was observed that more than half of the surveyed soldiers with obesity, regardless of whether it was diagnosed according to the criterion of BMI or percent body fat, had total cholesterol and triglyceride concentrations outside the normal range. This was also true for the IR-HOMA index, indicating insulin resistance. There was a particularly disturbing high percentage (61.3%) of those with elevated blood TG levels in the group with body fat above 25% compared to those with normal body fat (24.2%). 

## 4. Discussion

This study focused on body composition and cardiovascular health parameters of military flying personnel. The results showed that a significant percentage of soldiers were overweight or obese based on BMI, percent body fat, and waist circumference. However, the data showed that the prevalence of normal weight in this group heavily depends on the method used for diagnosis, likely due to so-called muscular overweight [24,26]. The prevalence of obesity, except for abdominal obesity, was affected to a lesser degree. Those diagnosed with obesity had higher levels of cholesterol, triglycerides, LDL-C, insulin, and HOMA-IR. There were also significant correlations between BMI, body fat percentage, waist circumference, and adverse blood test results. In addition, 8.5% of the soldiers surveyed met the criteria for metabolic syndrome. 

The findings highlight the importance of addressing obesity and its associated health risks among military personnel. Obesity can be viewed as a multifactorial pathology and chronic inflammatory disease [39]. In fact, people affected by obesity have a higher risk of developing interdependence and morbidity relative to healthy people. Obesity promotes the development of more than 200 chronic diseases [40]. In people with moderate obesity, the incidence of diabetes is ten times higher, and it is thirty times higher when body weight exceeds 135% of ideal weight. For cardiovascular diseases, such as high blood pressure, dyslipidemias, and coronary artery disease, there is a threefold increase in the risk of disease for obese people compared to those of normal weight [39]. Therefore, there is growing interest worldwide in early diagnosis of obesity to avoid the consequences of under-diagnosis or pre-diagnosis. 

The most commonly used parameter in diagnosis, the body mass index (BMI), is not suitable for assessing percent body fat. The main limitation of BMI, as mentioned in the introduction, is that it is impossible to distinguish BF from lean mass and central fat from peripheral fat [41]. This is especially important for military personnel, who are often found to have greater skeletal muscle mass than the general population [42]. In fact, many studies prove that BMI alone cannot define obesity, which consists not so much of weight gain as excess fat mass [43]. This was also confirmed by our own research, where the prevalence of overweight and obesity in the surveyed soldiers differed significantly depending on the classification of the assessment. Hence, it is recommended that soldiers should always have their body composition and waist circumference measured when assessing their nutritional status, in addition to calculating their BMI, in order to determine their actual body fat content.

Similar observations were made by De Lorenzo et al. [44] in comparing obesity classification according to BMI with classification according to percent body fat (25% for men and 30% for women), showing a large discrepancy between the two measurements. Even greater discrepancies according to the criterion adopted for diagnosing obesity were observed in a study of an adult population in central-southern Italy (n = 3258). According to BMI, 32.3% of the subjects were obese, while according to the acceptable percentage of fat mass as a function of gender and age, 64% of the population was characterized by obesity [41]. However, the authors noted that the false-negative BMI classification was stronger for women than men and for younger than older individuals.

The results of this study also indicate high prevalence of lipid disorders among Polish military flying personnel (48.5% of the subjects had elevated total cholesterol), which is consistent with unfavorable trends in the general population. Depending on the study sample selection, the prevalence of dyslipidemia in Poland is estimated at 60–80% of individuals in a population over 18 years of age [45]. Lipid metabolism disorders are still the most prevalent and least controlled risk factor for cardiovascular disease in Poland [46]. Together with smoking habits, type 2 diabetes, hypertension, poor dietary habits, and insufficient physical activity [7], as well as resulting overweight and obesity, they are among the main modifiable risk factors for atherosclerosis and its major complications, such as ischemic heart disease, stroke, and peripheral artery disease [47]. As indicated by the results of available epidemiological studies conducted in Poland, the prevalence of these diseases is steadily increasing, which is related to the spread of unfavorable eating habits, sedentary lifestyles, and the resulting obesity epidemic [48].

Our study found a significant association between soldiers’ obesity and carbohydrate–lipid disorders. The subjects with diagnosed obesity had significantly higher levels of biochemical parameters, such as total cholesterol, triglycerides, LDL-C, insulin levels, and HOMA-IR index, and lower HDL-C levels than participants at normal weight. In the US military, similar trends are observed [49]. These disturbing changes are evident even at a younger age. Although the primary goal of any armed forces is to prepare fit, combat-ready soldiers, the increase in CVD risk is increasingly evident in the military population. US Army soldiers are affected by cardiovascular disease more than any other chronic disease [50]. Similar findings were reported in Polish Armed Forces [18], where elevated total cholesterol was reported in more than half of the Polish soldiers surveyed, and the prevalence of abnormal low-density lipoprotein cholesterol was 60%. Furthermore, triglycerides were elevated in 36% of soldiers, and low high-density lipoprotein cholesterol and hyperglycemia were reported in 13% and 16% of soldiers, respectively. In the subgroup >50 years old, high and very high cardiovascular risk scores were observed in almost one third of soldiers. The relative risk assessed in younger subgroups was moderate or high. In our study, we obtained similar values to those cited. Elevated total cholesterol affected 48% of the soldiers studied and elevated triglycerides affected 35.5% of the subjects, while low high-density lipoprotein cholesterol and hyperglycemia were observed in 9% and 28% of the soldiers, respectively. In contrast, we observed half the percentage of military flying personnel with abnormal LDL-C values, which was at 38.7% (Figure 3, Figure 4). Analysis of the study results showed a significant increase in the percentage of those with TG concentrations above the desired value in soldiers with obesity, regardless of the diagnosis criterion. 

The health situation is much more favorable in the French army, where among the 1589 soldiers surveyed, less than 5% of the population reported a diagnosis of comorbidities: diabetes 2%, high blood pressure 1%, and dyslipidemia 1.5% [3]. 

The large number of overweight soldiers with unfavorable body fat distribution observed in our study group is worrisome, because in light of current data, obesity of the visceral type is an independent and significant risk factor for atherosclerosis and its complications, hypertension, and type II diabetes, among others. One of the direct consequences of visceral obesity is insulin resistance. The result of reduced tissue sensitivity to insulin is hyperglycemia and lipid disorders (mainly hypertriglyceridemia). Increased insulin secretion by pancreatic islet beta cells gradually leads to their exhaustion and the development of pre-diabetic states and diabetes. Hyperglycemia, lipid disorders, and hyperinsulinemia causing endothelial dysfunction and vascular wall remodeling accelerate the development of atherosclerosis and the onset of its clinical consequences [51]. In the study presented here, we observed an alarmingly high percentage of soldiers in the obese group with HOMA-IR above 2.5, which was more than 50%. This raises the question of whether the measurement of blood insulin levels should not be included in mandatory medical certification tests along with glucose levels in order to be able to prevent the development of diabetes earlier, albeit in a group of obese soldiers.

Military flying personnel, due to the tasks they perform, should be characterized by impeccable health, which, unfortunately, our study did not confirm. A prescription for existing metabolic disorders in the military environment could be the dissemination of the Mediterranean diet as a recommended dietary model. This is because this diet has many health benefits [52]. Studies conducted to date have also shown that the introduction of the Mediterranean diet resulted in a decrease in body weight and less frequent occurrence of metabolic syndrome in this group of patients, and it appears to be effective in both the prevention and treatment of cardiovascular disease and type 2 diabetes [53,54,55].

## 5. Limitations

As this is a cross-sectional observational study, is was only possible to elucidate associations between various measures of obesity and risks for CVD (including insulin resistance). Future follow-up studies will allow us to evaluate causal relationships between these measures in this cohort. Additionally, we did not measure the levels of spontaneous physical activity or diet of the studied soldiers, which are factors known to counteract the development of metabolic disorders. This will also be taken into account in further studies.

## 6. Conclusions

The findings suggest that the prevalence of obesity in Polish military flying personnel, regardless of the evaluation criterion, is associated with significant metabolic complications in the form of hyperlipidemia and insulin resistance, all of which are risk factors for cardiovascular disease. Significant differences in blood glucose concentrations were noted only between groups divided according to waist circumference. Yet BMI alone should not define obesity, which is particularly important in the case of military personnel, who are often found to have greater skeletal muscle mass than in the general population. Hence, it is recommended to include in the assessment of the nutritional status of soldiers routine body composition measurement tests in order to avoid overestimation or underestimation of obese individuals in this professional group. 

Prevention programs aimed at early assessment and modification of cardiovascular risk factors in the military flying personnel population are sorely needed, especially those aimed at weight reduction. In addition, the outcome of this study suggests that measurement of blood insulin levels should be included in mandatory medical certification examinations, along with glucose levels, to counteract the development of diabetes.

## Figures and Tables

**Table 1:** Results of anthropometric measurements and body composition analysis of subjects divided into groups according to BMI (N = 200).

Variables	Total (N = 200)			Normal (N = 73)		Overweight (N = 92)		Obese (N = 35)		*p*-Value
	X (SD)	Me	Min–Max	X (SD)	Me	X (SD)	Me	X (SD)	Me	
Age (years)	37.8 (8.5)	38.0	23.0–59.0	34.6 (8.3)	32.0	38.3 (8.0)	38.0	43.3 (7.0)	43.0	**<0.001**
Height (cm)	178.2 (6.4)	178.0	156.0–193.0	177.0 (7.5)	178.0	179.2 (5.9)	180.0	177.9 (4.5)	177.5	0.118
Weight (kg)	85.1 (13.4)	85.5	53.9–125.2	72.9 (8.9)	73.9	87.9 (6.9)	87.5	103.2 (8.8)	102.2	**<0.001**
BMI (kg/m^2^)	26.7 (3.7)	26.6	19.1–40.0	23.2 (1.5)	23.5	27.3 (1.3)	27.3	32.6 (2.8)	31.5	**<0.001**
TBW (kg)	49.1 (6.4)	49.9	31.2–64.0	44.7 (6.2)	46.3	50.9 (4.9)	50.6	53.6 (4.4)	53.8	**<0.001**
FM (kg)	18.2 (7.8)	16.5	5.8–52.0	12.0 (2.8)	11.5	18.5 (4.4)	18.5	30.2 (7.3)	28.6	**<0.001**
PBF (%)	20.8 (6.2)	20.6	8.1–41.6	16.6 (3.9)	16.8	21.0 (4.7)	21.3	29.0 (5.2)	28.4	**<0.001**
Visceral fat	7.4 (3.4)	7.0	1.0–20.0	4.5( 1.6)	4.0	7.7 (2.1)	8.0	12.5 (2.5)	12.0	**<0.001**
FFM (kg)	66.9 (8.7)	68.0	42.5–87.2	60.9 (8.5)	63.3	69.4 (6.7)	68.9	73.1 (6.0)	73.2	**<0.001**
SMM (kg)	38.2 (5.2)	38.7	23.3–50.0	34.5 (5.1)	35.9	39.7 (4.0)	39.4	41.8 (3.6)	41.6	**<0.001**
WC (cm)	93.4 (10.8)	93.0	63.0–125.0	83.7 (6.0)	84.0	95.8 (5.6)	95.0	107.2 (10.2)	108.0	**<0.001**
HC (cm)	101.0 (9.6)	100.8	62.0–193.0	95.2 (4.5)	96.0	103.1 (10.4)	103.0	107.9 (9.0)	109.0	**<0.001**
WHR	0.9 (0.1)	0.9	0.5–1.1	0.9 (0.1)	0.9	0.9 (0.1)	0.9	1.0 (0.0)	1.0	**<0.001**
WHtR	0.52 (0.06)	0.52	0.35–0.72	0.47 (0.03)	0.47	0.53 (0.03)	0.54	0.60 (0.06)	0.60	**<0.001**

N—group size, X—mean value, SD—standard deviation, Me—median, Min—minimum, Max—maximum, *p*—Kruskal–Wallis test statistical significance value. Bold values denote statistical significance at the *p* < 0.05 level. BMI—body mass index, TBW—total body water, FM—fat mass, PBF—percent of body fat, FFM—fat-free mass, SMM—skeletal muscle mass, WC—waist circumference, HC—hip circumference, WHR—waist–hip ratio, WHtR—waist-to-height ratio.

**Table 2:** Results of anthropometric measurements and body composition analysis of subjects divided into groups according to percent body fat (N = 200).

Variables	Total (N = 200)			Normal weight (N = 95)		Overweight (N = 74)		Obesity (N = 31)		*p*-Value
	X (SD)	Me	Min–Max	X (SD)	Me	X (SD)	Me	X (SD)	Me	
Age (years)	37.8 (8.5)	38.0	23.0–59.0	36.5 (8.4)	37.0	38.4 (8.70)	38.0	40.4 (7.7)	41.0	0.062
Height (cm)	178.2 (6.4)	178.0	156.0–193.0	179.4 (6.4)	178.5	176.9 (6.7)	177.3	177.6 (4.7)	177.0	0.056
Weight (kg)	85.1 (13.4)	85.5	53.9–125.2	79.6 (10.4)	79.4	85.9 (12.8)	86.5	100.0 (11.1)	97.9	**<0.001**
BMI (kg/m^2^)	26.7 (3.7)	26.6	19.1–40.0	24.7 (2.3)	24.7	27.3 (2.9)	27.4	31.7 (3.7)	30.8	**<0.001**
TBW (kg)	49.1 (6.4)	49.9	31.2–64.0	49.1 (6.4)	49.6	48.5 (7.0)	49.2	50.5 (4.3)	50.8	0.403
FM (kg)	18.2 (7.8)	16.5	5.8–52.0	12.7 (3.3)	12.4	19.8 (4.0)	19.5	31.2 (7.1)	28.9	**<0.001**
PBF (%)	20.8 (6.2)	20.6	8.1–41.6	15.9 (3.2)	15.9	22.9 (2.3)	22.8	30.9 (3.9)	29.6	**<0.001**
Visceral fat	7.4 (3.4)	7.0	1.0–20.0	4.9 (1.9)	5.0	8.2 (2.1)	8.0	12.8 (2.5)	12.0	**<0.001**
FFM (kg)	66.9 (8.7)	68.0	42.5–87.2	66.9 (8.7)	67.7	66.1 (9.5)	67.2	68.9 (5.8)	68.9	0.403
SMM (kg)	38.2 (5.2)	38.7	23.3–50.0	38.2 (5.3)	38.6	37.7 (5.7)	38.5	39.2 (3.5)	39.1	0.539
WC (cm)	93.4 (10.8)	93.0	63.0–125.0	87.8 (6.9)	88.0	95.4 (9.6)	96.0	105.8 (11.2)	105.0	**<0.001**
HC (cm)	101.0 (9.6)	100.8	62.0–193.0	98.1 (5.3)	98.0	102.5 (12.)	103.0	106.5 (9.7)	107.0	**<0.001**
WHR	0.9 (0.1)	0.9	0.8–1.2	0.9 (0.0)	0.9	0.9 (0.0)	0.9	1.0 (0.1)	1.0	**<0.001**
WHtR	0.52 (0.06)	0.52	0.35–0.72	0.49 (0.03)	0.49	0.54 (0.05)	0.55	0.60 (0.07)	0.59	**<0.001**

N—group size, X—mean value, SD—standard deviation, Me—median, Min—minimum, Max—maximum, *p*—Kruskal–Wallis test statistical significance value. Bold values denote statistical significance at the *p* < 0.05 level. BMI—body mass index, TBW—total body water, FM—fat mass, PBF—percent of body fat, FFM—fat-free mass, SMM—skeletal muscle mass, WC—waist circumference, HC—hip circumference, WHR—waist–hip ratio, WHtR—waist-to-height ratio.

**Table 3:** Results of laboratory blood tests of military flying personnel by group according to BMI (N = 200).

Variables	Total (N = 200)			Normal (N = 73)		Overweight (N = 92)		Obesity (N = 35)		*p*-Value
	X (SD)	Me	Min–Max	X (SD)	Me	X (SD)	Me	X (SD)	Me	
Ghrelin (ng/mL)	6.0 (4.3)	6.9	0.5–15.2	6.0 (4.4)	7.8	6.3 (4.3)	7.6	4.8 (3.9)	4.9	0.138
Glucose (mg/dL)	95.4 (9.6)	95.0	74.0–136.0	93.7 (9.0)	93.0	95.3 (9.2)	94.0	99.0 (11.2)	97.0	0.077
TC (mg/dL)	191.5 (32.7)	188.0	120.0–276.0	184.2 (31.0)	180.0	194.3 (32.5)	193.0	199.6 (34.5)	191.0	**0.044**
TG (mg/dL)	138.5 (82.3)	114.5	43.0–645.0	109.8 (51.7) **^###^	92.0	147.9 (87.2) **	121.5	173.7 (101.7) ^###^	150.0	**<0.001**
LDL-C (mg/dL)	109.1 (28.9)	106.0	8.0–190.0	102.1 (27.4) *^#^	98.0	112.1 (29.5) *^#^	112.0	115.8 (28.3) ^#^	112.0	**0.014**
HDL-C (mg/dL)	55.3 (12.7)	53.5	33.0–96.0	60.2 (12.4) **^###^	59.0	53.8 (12.2) **	51.0	49.1 (11.0) ^###^	49.0	**<0.001**
Insulin (mIU/L)	8.4 (3.9)	7.5	0.4–27.7	6.8 (2.5) *^###^	6.9	8.2 (2.8) *^$$^	7.6	12.1 (5.8) ^###$$^	12.2	**<0.001**
HOMA-IR	2.0 (1.0)	1.8	0.1–6.6	1.6 (0.6) *^###^	1.6	2.0 (0.7) *^$$^	1.8	3.0 (1.4) ^###$$^	3.3	**<0.001**

N—group size, X—mean value, SD—standard deviation, Me—median, Min—minimum, Max—maximum, *p*—Kruskal–Wallis test statistical significance value. Bold values denote statistical significance at the *p* < 0.05 level. TC—total cholesterol, TG—triglyceride, LDL-C—low-density lipoprotein cholesterol, HDL-C—high-density lipoprotein cholesterol, * Normal vs. Overweight; *p* < 0.05, ** Normal vs. Overweight; *p* < 0.01, ^#^ Normal vs. Obese; *p* < 0.05, ^###^ Normal vs. Obese; *p* < 0.001, ^$$^ Overweight vs. Obese; *p* < 0.01.

**Table 4:** Results of laboratory blood tests of military flying personnel by group according to percent body fat content (N = 200).

Variables	Total (N = 200)			Normal (N = 95)		Overweight (N = 74)		Obesity (N = 31)		*p*-Value
	X (SD)	Me	Min–Max	X (SD)	Me	X (SD)	Me	X (SD)	Me	
Ghrelin (ng/mL)	6.0 (4.3)	6.9	0.5–15.2	6.1 (4.6)	6.9	6.0 (4.0)	6.9	5.4 (4.2)	5.6	<0.913
Glucose (mg/dL)	95.4 (9.6)	95.0	74.0–136.0	94.7 (8.6)	93.0	94.7 (8.7)	95.0	99.0(13.4)	95.0	<0.429
TC (mg/dL)	191.5 (32.7)	188.0	120.0–276.0	188.2 (32.7)	186.0	192.9 (32.7)	188.5	198.4 (32.4)	197.0	<0.234
TG (mg/dL)	138.5 (82.3)	114.5	43.0–645.0	121.4 (83.3) *^###^	100.0	144.7 (79.7) *	120.0	176.1 (72.3) ^###^	168.0	**<0.001**
LDL-C (mg/dL)	109.1 (28.9)	106.0	8.0–190.0	107.0 (28.6)	103.5	109.2 (30.4)	112.0	115.1 (26.3)	112.0	<0.275
HDL-C (mg/dL)	55.3 (12.7)	53.5	33.0–96.0	58.0 (12.5) ^###^	57.0	54.9 (12.9) ^$^	53.0	48.1 (10.0) ^###$^	47.0	**<0.001**
Insulin (mIU/L)	8.4 (3.9)	7.5	0.4–27.7	7.1 (3.0) *^###^	6.8	8.4 (2.8) *^$$^	7.7	12.2 (5.8) ^###$$^	11.1	**<0.001**
HOMA-IR	2.0 (1.0)	1.8	0.1–6.6	1.7(0.7) *^###^	1.6	2.0 (0.8) *^$$^	1.8	3.0 (1.4) ^###$$^	2.6	**<0.001**

N—group size, X—mean value. SD—standard deviation, Me—median, Min—minimum, Max—maximum, *p*—Kruskal–Wallis test statistical significance value. Bold values denote statistical significance at the *p* < 0.05 level. TC—total cholesterol, TG—triglyceride, LDL-C—low-density lipoprotein cholesterol, HDL-C—high-density lipoprotein cholesterol, * Normal vs. Overweight; *p* < 0.05, ^###^ Normal vs. Obese; *p* < 0.001, ^$^ Overweight vs. Obese; *p* < 0.05, ^$$^ Overweight vs. Obese; *p* < 0.01.

**Table 5:** Results of laboratory blood tests of military flying personnel by group according to waist circumference (N = 200).

Variables	Total (N = 200)			Waist Circumference up 94 cm (N = 106)		Waist Circumference 94–102 cm (N = 53)		Waist Circumference > 102 cm (N = 41)		*p*-Value
	M (SD)	Me	Min–Max	X (SD)	Me	X (SD)	Me	X (SD)	Me	
Ghrelin (ng/mL)	6.0 (4.3)	6.9	0.5–15.2	6.0 (4.4)	7.2	6.4 (4.2)	7.5	5.1 (4.1)	4.9	0.612
Glucose (mg/dL)	95.4 (9.6)	95.0	74.0–136.0	93.7 (9)	93.0	96.7 (7.5)	96.0	98.2 (12.5)	96.0	**0.048**
TC (mg/dL)	191.5 (32.7)	188.0	120.0–276.0	184.9 (31.1) *	182	200.5 (36.1) *	193.0	197.1 (28.6)	194.0	**0.017**
TG (mg/dL)	138.5 (82.3)	114.5	43.0–645.0	114.4 (54.7) *^###^	98.0	154.5 (108.1) *	127.0	180.2 (83.5) ^###^	176.0	**<0.001**
LDL-C (mg/dL)	109.1 (28.9)	106.0	8.0–190.0	103.0 (28.2) *	101.5	118.3 (31.1) *	115.0	114 (25.4)	114.0	**<0.001**
HDL-C (mg/dL)	55.3 (12.7)	53.5	33.0–96.0	59 (12.8) *^###^	58.0	53.7 (11.4) *	53.0	47.8 (10.5) ^###^	46.0	**<0.001**
Insulin (mIU/L)	8.4 (3.9)	7.5	0.4–27.7	7.0(2.6) ***^###^	6.8	9.2 (3.8) ***	8.4	11.1 (5.0) ^###^	9.8	**<0.001**
HOMA-IR	2.0 (1.0)	1.8	0.1–6.6	1.6 (0.7) ***^###^	1.6	2.2 (0.9) ***	2.1	2.7 (1.2) ^###^	2.4	**<0.001**

N—group size, M—mean value, SD—standard deviation, Me—median, Min—minimum, Max—maximum, *p*—Kruskal-Wallis test statistical significance value, Bold values denote statistical significance at the *p* < 0.05 level, TC—total cholesterol, TG—triglyceride, LDL-C—low-density lipoprotein cholesterol, HDL-C—high-density lipoprotein cholesterol, * Normal vs. Overweight; *p* < 0.05, *** Normal vs. Overweight; *p* < 0.001, ^###^ Normal vs. Obese; *p* < 0.001.

**Table 6:** Correlations between BMI, percent body fat, and body circumference and WHtR and blood parameters of military flying personnel (N = 200).

Variables	BMI (kg/m^2^)		Percent Body Fat (%)		Waist Circumference (cm)		WHtR	
	R	P	R	P	R	P	R	P
Ghrelin (ng/mL)	−0.07	0.34	−0.09	0.22	−0.02	0.78	−0.03	0.67
Glucose (mg/dL)	0.20	**0.005**	0.20	**0.005**	0.20	**0.004**	0.24	**0.0005**
TC (mg/dL)	0.18	**0.01**	0.17	**0.02**	0.19	**0.008**	0.24	**0.0007**
TG (mg/dL)	0.39	**<0.0001**	0.39	**<0.0001**	0.39	**<0.0001**	0.44	**<0.0001**
LDL-C (mg/dL)	0.20	**0.004**	0.14	**0.05**	0.21	**0.003**	0.24	**<0.0001**
HDL-C (mg/dL)	−0.40	**<0.0001**	−0.30	**<0.0001**	−0.42	**<0.0001**	−0.42	**<0.0001**
Insulin (mIU/L)	0.42	**<0.0001**	0.45	**<0.0001**	0.45	**<0.0001**	0.46	**<0.0001**
HOMA-IR	0.43	**<0.0001**	0.45	**<0.0001**	0.46	**<0.0001**	0.48	**<0.0001**

N—group size, R—Spearman correlations, *p*—level of statistical significance. Bold values denote statistical significance at the *p* < 0.05 level. TC—total cholesterol, TG—triglyceride, LDL-C—low-density lipoprotein cholesterol, HDL-C—high-density lipoprotein cholesterol.

## Data Availability

Data sharing is not applicable to this article.

## References

[1] Afshin A., Forouzanfar M.H., Reitsma M.B., Sur P., Estep K., Lee A., Marczak L., Mokdad A.H., Moradi-Lakeh M., Naghavi M. (2017). Health Effects of Overweight and Obesity in 195 Countries over 25 Years. N. Engl. J. Med..

[2] Hruby A., Hill O.T., Bulathsinhala L., McKinnon C.J., Montain S.J., Young A.J., Smith T.J. (2015). Trends in Overweight and Obesity in Soldiers Entering the US Army, 1989–2012. Obesity.

[3] Quertier D., Goudard Y., Goin G., Regis-Marigny L., Sockeel P., Dutour A., Pauleau G., De La Villeon B. (2022). Overweight and Obesity in the French Army. Mil. Med..

[4] Sanderson P.W., Clemes S.A., Biddle S.J.H. (2014). Prevalence and socio-demographic correlates of obesity in the British Army. Ann. Hum. Biol..

[5] Salimi Y., Taghdir M., Sepandi M., Zarchi A.A.K. (2019). The prevalence of overweight and obesity among Iranian military personnel: A systematic review and meta-analysis. BMC Public Health.

[6] Gaździńska A., Jagielski P., Baran P. (2018). Evaluation of nutritional status and the level of physical fitness of military flying personnel staying at the training camp. Pol. J. Aviat. Med. Bioeng Psychol..

[7] Gazdzinska A., Jagielski P., Turczynska M., Dziuda L., Gazdzinski S. (2022). Assessment of Risk Factors for Development of Overweight and Obesity among Soldiers of Polish Armed Forces Participating in the National Health Programme 2016–2020. Int. J. Environ. Res. Public Health.

[8] Gazdzinska A., Baran P., Skibniewski F., Truszczynski O., Gazdzinski S., Wylezol M. (2015). The prevalence of overweight and obesity vs. the level of physical activity of aviation military academy students. Med. Pr..

[9] Al-Qahtani D.A., Imtiaz M.L., Shareef M.M. (2005). Obesity and cardiovascular risk factors in Saudi adult soldiers. Saudi Med. J..

[10] Williams E.P., Mesidor M., Winters K., Dubbert P.M., Wyatt S.B. (2015). Overweight and Obesity: Prevalence, Consequences, and Causes of a Growing Public Health Problem. Curr. Obes. Rep..

[11] Hu L.H., Huang X., You C.J., Li J.X., Hong K., Li P., Wu Y.Q., Wu Q.H., Wang Z.W., Gao R.L. (2017). Prevalence of overweight, obesity, abdominal obesity and obesity-related risk factors in southern China. PLoS ONE.

[12] Alpert M.A. (2001). Obesity cardiomyopathy: Pathophysiology and evolution of the clinical syndrome. Am. J. Med. Sci..

[13] Lavie C.J., Milani R.V., Ventura H.O. (2009). Obesity and Cardiovascular Disease Risk Factor, Paradox, and Impact of Weight Loss. J. Am. Coll. Cardiol..

[14] Lavie C.J., McAuley P.A., Church T.S., Milani R.V., Blair S.N. (2014). Obesity and Cardiovascular Diseases. J. Am. Coll. Cardiol..

[15] Jamaluddin M.S., Weakley S.M., Yao Q.Z., Chen C.Y. (2012). Resistin: Functional roles and therapeutic considerations for cardiovascular disease. Br. J. Pharmacol..

[16] Abate N., Sallam H.S., Rizzo M., Nikolic D., Obradovic M., Bjelogrlic P., Isenovic E.R. (2014). Resistin: An Inflammatory Cytokine. Role in Cardiovascular Diseases, Diabetes and the Metabolic Syndrome. Curr. Pharm. Des..

[17] Laakso M. (2015). Is Insulin Resistance a Feature of or a Primary Risk Factor for Cardiovascular Disease?. Curr. Diab. Rep..

[18] Gielerak G., Krzesinski P., Piotrowicz K., Murawski P., Skrobowski A., Stanczyk A., Galas A., Uzieblo-Zyczkowska B., Kazmierczak-Dziuk A., Maksimczuk J. (2020). The Prevalence of Cardiovascular Risk Factors among Polish Soldiers: The Results from the MIL-SCORE Program. Cardiol. Res. Pract..

[19] Gielerak G., Krzesiński P., Stańczyk A. (2013). Cardiovascular risk factors among soldiers-candidates for service abroad. The new perspective of epidemiological studies and pro-health behaviors in general population of the armed forces. Lekarz Wojskowy.

[20] Chait A., den Hartigh L.J. (2020). Adipose Tissue Distribution, Inflammation and Its Metabolic Consequences, Including Diabetes and Cardiovascular Disease. Front. Cardiovasc. Med..

[21] Gruzdeva O., Borodkina D., Uchasova E., Dyleva Y., Barbarash O. (2018). Localization of fat depots and cardiovascular risk. Lipids Health Dis..

[22] Garcia A.S.E., Moreno A.G.M., Castillo Z.R. (2021). The role of ghrelin and leptin in feeding behavior: Genetic and molecular evidence. Endocrinol. Diabetes Y Nutr..

[23] Obesity and Overweight Factsheet from the WHO. https://www.who.int/news-room/fact-sheets/detail/obesity-and-overweight.

[24] Tomczak A., Bertrandt J., Kłos A., Bertrandt B. (2014). Assessment of physical fitness, physical capacity and nutritional status of soldiers serving in the “GROM” Polish Special Forces Unit. Probl. Hig. Epidemiol..

[25] Tomczak A. (2012). Physical activity of soldiers in the Polish Armed Force’s military administration units and special units. Biomed. Hum. Kinet..

[26] Zhu Q.Q., Huang B.B., Li Q.L., Huang L.Q., Shu W.B., Xu L., Deng Q.Y., Ye Z.L., Li C.Y., Liu P. (2020). Body mass index and waist-to-hip ratio misclassification of overweight and obesity in Chinese military personnel. J. Physiol. Anthropol..

[27] Son Y.J., Kim J., Park H.J., Park S.F., Park C.Y., Lee W.Y., Oh K.W., Park S.W., Rhee E.J. (2016). Association of Waist-Height Ratio with Diabetes Risk: A 4-Year Longitudinal Retrospective Study. Endocrinol. Metab..

[28] Browning L.M., Hsieh S.D., Ashwell M. (2010). A systematic review of waist-to-height ratio as a screening tool for the prediction of cardiovascular disease and diabetes: 0.5 could be a suitable global boundary value. Nutr. Res. Rev..

[29] Khan R.J., Harvey D.J., Leistikow B.N., Haque K.S., Stewart C.P. (2015). Relationship between obesity and coronary heart disease among urban Bangladeshi men and women. Integr. Obes. Diabetes.

[30] Jablonowska-Lietz B., Wrzosek M., Wlodarczyk M., Nowicka G. (2017). New indexes of body fat distribution, visceral adiposity index, body adiposity index, waist-to-height ratio, and metabolic disturbances in the obese. Kardiol. Pol..

[31] Ashwell M., Hsieh S.D. (2005). Six reasons why the waist-to-height ratio is a rapid and effective global indicator for health risks of obesity and how its use could simplify the international public health message on obesity. Int. J. Food Sci. Nutr..

[32] Brończyk-Puzoń A., Koszowska A., Bieniek J. (2018). Basic anthropometric measurements and derived ratios in dietary counseling: Part one. Nurs. Public Health.

[33] Gallagher D., Heymsfield S.B., Heo M., Jebb S.A., Murgatroyd P.R., Sakamoto Y. (2000). Healthy percentage body fat ranges: An approach for developing guidelines based on body mass index. Am. J. Clin. Nutr..

[34] Alberti K., Zimmet P., Shaw J. (2006). Metabolic syndrome—A new world-wide definition. A consensus statement from the international diabetes federation. Diabet. Med..

[35] Yoon Y.S., Oh S.W. (2014). Optimal Waist Circumference Cutoff Values for the Diagnosis of Abdominal Obesity in Korean Adults. Endocrinol. Metab..

[36] Lean M.E.J., Han T.S., Morrison C.E. (1995). Waist circumference as a measure for indicating need for weight management. Br. Med. J..

[37] Yoshitomi Y., Ishii T., Kaneki M., Tsujibayashi T., Sakurai S.I., Nagakura C., Miyauchi A. (2005). Relationship between insulin resistance and effect of atorvastatin in non-diabetic subjects. J. Atheroscler. Thromb..

[38] Dobrowolski P., Prejbisz A., Kuryłowicz A., Burchardt P., Chlebus K. (2022). Zespół metaboliczny—Nowa definicja i postępowanie w praktyce. Nadciśnienie Tętnicze Prakt..

[39] Vallgarda S., Nielsen M.E.J., Hansen A.K.K., Cathaoir K.O., Hartlev M., Holm L., Christensen B.J., Jensen J.D., Sorensen T.I.A., Sandoe P. (2017). Should Europe follow the US and declare obesity a disease?: A discussion of the so-called utilitarian argument. Eur. J. Clin. Nutr..

[40] Jastreboff A.M., Kotz C.M., Kahan S., Kelly A.S., Heymsfield S.B. (2019). Obesity as a Disease: The Obesity Society 2018 Position Statement. Obesity.

[41] De Lorenzo A., Bianchi A., Maroni P., Iannarelli A., Di Daniele N., Iacopino L., Di Renzo L. (2013). Adiposity rather than BMI determines metabolic risk. Int. J. Cardiol..

[42] Tomczak A., Anyzewska A., Bertrandt J., Lepionka T., Kruszewski A., Gazdzinska A. (2022). Assessment of the Level of Physical Activity and Body Mass Index of Soldiers of the Polish Air Force. Int. J. Environ. Res. Public Health.

[43] De Lorenzo A., Gratteri S., Gualtieri P., Cammarano A., Bertucci P., Di Renzo L. (2019). Why primary obesity is a disease?. J. Transl. Med..

[44] De Lorenzo A., Deurenberg P., Pietrantuono M., Di Daniele N., Cervelli V., Andreoli A. (2003). How fat is obese?. Acta Diabetol..

[45] Jóźwiak J., Windak A., Mastalerz-Migas A., Chlabicz S. (2015). Dyslipidemie. Medycyna Rodzinna. Podręcznik Dla Lekarzy i Studentów.

[46] Banach M., Jankowski P., Jozwiak J., Cybulska B., Windak A., Guzik T., Mamcarf A., Broncel M., Tomasik T., Rysz J. (2017). PoLA/CFPiP/PCS Guidelines for the Management of Dyslipidaemias for Family Physicians 2016. Arch. Med. Sci..

[47] Mach F., Baigent C., Catapano A.L., Koskinas K.C., Casula M., Badimon L., Chapman M.J., De Backer G.G., Delgado V., Ference B.A. (2020). 2019 ESC/EAS Guidelines for the management of dyslipidaemias: Lipid modification to reduce cardiovascular risk The Task Force for the management of dyslipidaemias of the European Society of Cardiology (ESC) and European Atherosclerosis Society (EAS). Eur. Heart J..

[48] Studzinski K., Tomasik T., Windak A., Banach M., Wojtowicz E., Mastej M., Tomaszewski M., Mikhailidis D.P., Toth P.P., Catapano A. (2021). The Differences in the Prevalence of Cardiovascular Disease, Its Risk Factors, and Achievement of Therapeutic Goals among Urban and Rural Primary Care Patients in Poland: Results from the LIPIDOGRAM 2015 Study. J. Clin. Med..

[49] McGraw L.K., Turner B.S., Stotts N.A., Dracup K.A. (2008). A review of cardiovascular risk factors in US military personnel. J. Cardiovasc. Nurs..

[50] Shrestha A., Ho T.E., Vie L.L., Labarthe D.R., Scheier L.M., Lester P.B., Seligman M.E.P. (2019). Comparison of Cardiovascular Health Between US Army and Civilians. J. Am. Heart Assoc..

[51] Bornfeldt K.E., Tabas I. (2011). Insulin Resistance, Hyperglycemia, and Atherosclerosis. Cell Metab..

[52] Sofi F., Macchi C., Abbate R., Gensini G.F., Casini A. (2014). Mediterranean diet and health status: An updated meta-analysis and a proposal for a literature-based adherence score. Public Health Nutr..

[53] Salas-Salvado J., Bullo M., Babio N., Martinez-Gonzalez M.A., Ibarrola-Jurado N., Basora J., Estruch R., Covas M.I., Corella D., Aros F. (2011). Reduction in the Incidence of Type 2 Diabetes with the Mediterranean Diet Results of the PREDIMED-Reus nutrition intervention randomized trial. Diabetes Care.

[54] Martinez-Gonzalez M.A., de la Fuente-Arrillaga C., Nunez-Cordoba J.M., Basterra-Gortari F.J., Beunza J.J., Vazquez Z., Benito S., Tortosa A., Bes-Rastrollo M. (2008). Adherence to Mediterranean diet and risk of developing diabetes: Prospective cohort study. BMJ Br. Med. J..

[55] Dinu M., Pagliai G., Casini A., Sofi F. (2018). Mediterranean diet and multiple health outcomes: An umbrella review of meta-analyses of observational studies and randomised trials. Eur. J. Clin. Nutr..

